# Sight threatening vitreous haemorrhage and retinal detachment in a patient with sickle cell disease

**DOI:** 10.11604/pamj.2020.35.1.17098

**Published:** 2020-01-02

**Authors:** Jemal Zeberga Shifa, Alemayehu Mekonnen Gezmu

**Affiliations:** 1 Department of Surgery, Medical Faculty, University of Botswana, Botswana; 2 Department of Paediatrics and Adolescent Health, Faculty of Medicine, University of Botswana, Botswana

**Keywords:** Sickle cell disease, vitreous haemorrhage, retinal detachment

## Abstract

We report a case of sight threatening vitreous haemorrhage and retinal detachment as complication of sickle cell disease (SCD). A 35 years old female Nigerian patient had presented to ophthalmology clinic of Princess Marina Hospital, Botswana, with two weeks history of poor vision in the left eye. The loss of vision was due to vitreous haemorrhage and retinal detachment which was confirmed by direct and indirect ophthalmoscopy and B-Scan ultrasound. Prior to presentation, patient didn't have any follow up by an ophthalmologist as part of regular medical care for patients with SCD. We emphasize the importance of regular follow up for early detection, treatment and prevention of complication associated with sickle cell disease.

## Introduction

Normal red blood cell haemoglobin comprises four polypeptide globin chains, each associated with a central hemering (ferroprotoporphyrin). The globin chains consist of two identical pairs of α and β polypeptide chains. The sickle-cell haemoglobinopathies are characterized by a genetic error in β-chain synthesis, which results in abnormal function of the haemoglobin molecule. In conditions of ischemia and metabolic stress, the imperfect globin chains induce pathologic alterations in red blood cell morphology. Owing to their crescent shape, the altered red blood cells are labelled ‘sickle cells’s [1]. The phenotypic heterogeneity of sickle cell disease (SCD) ranges from haemolytic anaemia and chronic vasculopathy with acute painful crises to a multitude of organ-specific manifestations of varying severity. The exact cause of this diversity of clinical features remains unclear, but recent advances in the understanding of clinical SCD indicate that the pathophysiology of this disease is complex and multifaceted. Factors that influence the disease process include genetic variation, the proportion of sickled cells, endothelial cell activation, inflammation, oxidative stress and hypoxia-induced angiogenesis [2,3]. Since many of the problems are time dependent, the overall prevalence of ocular complications of sickle disease is unknown. Although various systemic complications of SCD are known to be more common in patients with the Hb SS genotype, visual impairment secondary to proliferative sickle retinopathy is more common in patients with the Hb SC genotype. In a large cohort, 6% (49/783) of Hb SS genotype patients had proliferative sickle retinopathy (PSR) on initial examination compared with 32% (172/533) of Hb SC genotype patients [4]. Approximately 40-43% of haemoglobin Hb SC genotype patients and 14-20% of haemoglobin Hb SS patients were expected to develop PSR [5, 6].

## Patient and observation

**Figure 1:**
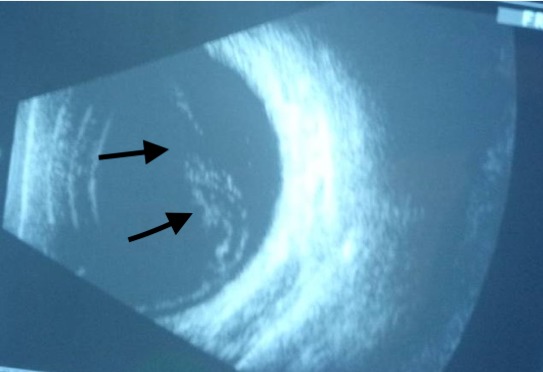
B scan ultrasound of the left eye with evidence of vitreous haemorrhage and retinal detachment (arrows)

A 35 years old female Nigerian patient was diagnosed to have Sickle cell disease since early child hood. She presented to our ophthalmology clinic, in Gaborone, Botswana, with reduction of vision in the left eye for two weeks. During initial evaluation, the visual acuity in right eye was 6/6 and in the left eye counting fingers from a distance of 5 meters. Intraocular pressure in both eyes was with in normal range. Posterior segment evaluation using direct and indirect ophthalmoscopy showed retinal detachment and vitreous haemorrhage in the left eye. B scan ultrasound showed vitreous opacity with retinal detachment in the left eye (Figure 1). The patient was followed in our clinic for more than 3 months with no improvement in her vision on the affected side. Decision was made to perform parsplana vitrectomy. However, the patient refused the surgery despite repeated counselling. Currently her vision on left eye is only light perception.

## Discussion

Nearly all ocular or periocular structures can be affected by the sickle haemoglobinopathies. Classic anterior segment findings include conjunctival ´comma-shaped´ capillaries which represent intravascular sludging of sickling red blood cells [7]. Sectorial iris atrophy represents areas of anterior uveal ischemia. A mild anterior chamber cell and flare reaction may be observed secondary to incompetence of the blood-ocular barrier. The anterior segment manifestations generally do not pose significant risks for vision loss. Posterior segment manifestations of sickle haemoglobinopathies may be observed in the vitreous body, optic disc, retina and sub retinal structures. Vitreous haemorrhage secondary to peripheral retinal neovascularization may develop. The optic disc may demonstrate sludging red blood cells within prepapillary retinal capillaries. More debilitating macular ischemia can result in frank macular infarction with an enlarged foveal avascular zone secondary to multiple retinal arteriolar occlusions with an often insidious, progressive course [8,9]. Our patient presented with advanced stage of ocular complication of SCD because of lack of awareness about the importance of regular ophthalmologic evaluation by the patient and allied health care professional for early identification of ocular complications of SCD.

## Conclusion

We emphasize the importance of ophthalmologic regular check-up for patients with SCD. Delay in diagnosis and management can lead to loss of vision as the result of ocular complication of sickle cell disease.

## Competing interests

The authors declare no competing interests.
